# Effects of atorvastatin on atrial remodeling in a rabbit model of atrial fibrillation produced by rapid atrial pacing

**DOI:** 10.1186/s12872-016-0301-8

**Published:** 2016-06-24

**Authors:** Qian Yang, Xiaoyong Qi, Yi Dang, Yingxiao Li, Xuelian Song, Xiao Hao

**Affiliations:** Department of Internal Medicine, Hebei Medical University, Shijiazhuang, Hebei Province People’s Republic of China; Department of Cardiology, Hebei General Hospital, Shijiazhuang, Hebei Province People’s Republic of China

**Keywords:** Atrial fibrillation, Atrial remodeling, Myeloperoxidase, Atorvastatin

## Abstract

**Background:**

Accumulating evidence suggests that myeloperoxidase (MPO) is involved in atrial remodeling of atrial fibrillation (AF). Statins could reduce the MPO levels in patients with cardiovascular diseases. This study evaluated the effects of atorvastatin on MPO level and atrial remodeling in a rabbit model of pacing-induced AF.

**Methods:**

Eighteen rabbits were randomly divided into sham, control and atorvastatin groups. Rabbits in the control and atorvastatin groups were subjected to rapid atrial pacing (RAP) at 600 bpm for 3 weeks, and treated with placebo or atorvastatin (2.5 mg/kg/d), respectively. Rabbits in the sham group did not receive RAP. After 3 weeks of pacing, atrial structural and functional changes were assessed by echocardiography, atrial effective refractory period (AERP) and AF inducibility were measured by atrial electrophysiological examination, and histological changes were evaluated by Masson trichrome-staining. The L-type calcium channel α1c (Cav1.2), collagen I and III, MPO, matrix metalloproteinase (MMP)-2 and MMP-9 were analyzed by real time polymerase chain reaction and/or western blot.

**Results:**

All rabbits were found to have maintained sinus rhythm after 3 weeks of RAP. Atrial burst stimulation induced sustained AF (>30 min) in 5, 4, and no rabbits in the control, atorvastatin, and sham groups, respectively. The AERP shortened and Cav1.2 mRNA level decreased in the control group, but these changes were suppressed in the atorvastatin group. Obvious left atrial enlargement and dysfunction was found in both control and atorvastatin groups. Compared with the control group, these echocardiograhic indices of left atrium did not differ in the atorvastatin group. Prominent atrial fibrosis and increased levels of collagen I and III were observed in the control group but not in the atorvastatin group. The mRNA and protein levels of MPO, MMP-2 and MMP-9 significantly increased in the control group, but these changes were prevented in the atorvastatin group.

**Conclusion:**

Treatment with atorvastatin prevented atrial remodeling in a rabbit model of RAP-induced AF. The reduction of levels of atrial MPO, MMP-2 and MMP-9 may contribute to the prevention of atorvastatin on atrial remodeling.

## Background

Atrial fibrillation (AF) is the most common sustained arrhythmia in clinical practice, increasing in prevalence with age [1]. It results in serious potential complications, especially stroke and heart failure, and increased mortality [2]. Despite recent advances in pharmacological strategy and radiofrequency ablation, the treatment of AF is still not satisfactory.

In recent years, accumulating evidence suggests that the main mechanisms contributing to the initiation and maintenance of AF are atrial electrical and structural remodeling [3, 4], and myeloperoxidase (MPO) is involved in the associated atrial remodeling [5, 6]. MPO is a major contributor to inflammatory oxidative stress, and catalyzes the generation of reactive species [6]. It is a crucial prerequisite for atrial fibrosis, leading to an increased vulnerability to AF [5]. Patients with AF had higher plasma and atrial MPO levels compared with individuals in sinus rhythm [5], and high MPO level predicted an increased risk of AF recurrence after catheter ablation [7].

Statins are widely used in primary and secondary prevention of ischemic heart disease and stroke, because of their lipid-lowering effect. In addition, statins also have anti-inflammatory and antioxidant properties, which may help prevent AF [8]. Recent meta-analyses showed that the use of statins is associated with a decreased risk of AF in patients with sinus rhythm [9, 10]. Some studies also reported that statins could attenuate atrial electrical or structural remodeling in dog and goat AF models [11–14]. However, the molecular mechanism by which statins may prevent AF has not been elucidated. Previous research showed that statins could reduce the MPO levels in patients with cardiovascular diseases [15, 16]. This study was designed to investigate the potential effects of atorvastatin on MPO level and atrial remodeling in a rabbit model of pacing-induced AF.

## Methods

### Animal preparation

Eighteen male New Zealand white rabbits (2.5–3.0 kg) were randomly allocated to sham (*n* = 6), control (*n* = 6) and atorvastatin (*n* = 6) groups. All rabbits were anesthetized with an intravenous injection of 3 % pentobarbital sodium (30 mg/kg). The left thoracic cavity was opened via 2–4 intercostals, and then the heart was exposed by a dilator. One thin silicon plaque containing two pairs of electrodes was implanted in the free wall of the left atrial appendage. One pair was connected to a pacemaker (Fudan University, Shanghai, China) implanted in a subcutaneous pocket on the back of the rabbit. The other pair was tunneled subcutaneously and exposed at the back of the rabbit, and used for electrophysiological measurements [17]. When surgery was completed, rabbits were given antibiotics and allowed to recover for one week. After that, rabbits in the control and atorvastatin groups were subjected to rapid atrial pacing (RAP) at 600 beats/min for 3 weeks, meanwhile they received oral placebo or atorvastatin (2.5 mg/kg/day), respectively. Rabbits in the sham group did not receive RAP.

### Electrophysiological study

Electrocardiogram (ECG) was recorded before and after the pacing. During the period of RAP, ECG was measured every day to ensure that the pacemakers were working properly.

The atrial effective refractory period (AERP) was measured at a basic cycle length of 150 ms. Eight basic stimuli (S1) were followed by a premature stimulus (S2). The S1-S2 intervals were decreased in 10 ms steps until S2 failed to produce an atrial response, then increased by 10 ms, and decreased in 2 ms steps until S2 capture failure. The longest S1-S2 interval that failed to capture was defined as the AERP_150_ [11, 17].

AF was induced with a train of 10Hz, 2 ms stimuli to the left atrium at four times threshold current [17] and was induced ten times in each rabbit. AF was considered sustained if it persisted for more than 30 min.

### Echocardiography

The structure and function of the left atrium (LA) and left ventricle (LV) were assessed by transthoracic echocardiographic examinations (Philips IU 22, Washington, USA). LV end diastolic diameter (LVEDD) and end systolic diameter (LVESD), LV ejection fraction (LVEF), LA diameter (LAD), LA maximal volume (LAV_max_) and minimal volume (LAV_min_) were measured before and after the pacing. The volume measurements were calculated from apical 4- and 2-chamber views using the biplane area-length method. LAV_max_ was recorded immediately before the mitral valve opening and LAV_min_ was recorded at mitral valve closure. LA ejection fraction (LAEF) was calculated according to the formula: (LAV_max_- LAV_min_)/LAV_max_ × 100 % [18].

### Histological examination

At the end of the experiments, all rabbits were euthanized and then the LA free wall tissues were quickly removed. Formalin-fixed paraffin-embedded tissues were stained with Masson’s trichrome. The collagen fibers were marked with blue, while the cardiomyocytes were marked with red. Fibrous tissue areas were quantified using Image Pro Plus 6.0 software (Media Cybernetics, Maryland, USA) [19].

### Western blot analysis

The total proteins were purified from the LA free wall, separated by 10 % SDS-PAGE, and then transferred onto a polyvinylidene difluoride membrane. This was blocked at room temperature for 1 h in Tris-buffered saline with 0.5 % Tween 20 containing 5 % skim milk and probed with primary antibodies overnight at 4 °C.

The following primary antibodies were independently used to detect specific proteins: collagen I (1:500 dilution, Bioworld, USA), collagen III (1:500 dilution, Bioworld, USA), MPO (1:200 dilution, Santa Cruz, USA), matrix metalloproteinase (MMP)-2 (1:500 dilution, ProteinTech, USA), MMP-9 (1:500 dilution, ProteinTech, USA), and tissue inhibitors of metalloproteinase (TIMP)-1 (1:1000 dilution, Abcam, USA). An antibody against β-actin (1:1000 dilution, ProteinTech, USA) was used as an internal control.

Horseradish peroxidase-conjugated anti-goat (1:10000 dilution, Beyotime, China) or anti-mouse (1:10000 dilution, ZSGB Biological Company, China) IgGs were used to bind the primary antibodies. Protein bands on Western blots were visualized using an enhanced chemiluminescence detection system (Santa Cruz, USA). Relative band densities of proteins in Western blots were normalized against β-actin.

### Real time polymerase chain reaction (RT-PCR)

Total RNA was extracted with TRIZOL reagent (Invitrogen, USA). In accordance with the protocol provided by the manufacturer, cDNA was synthesized with an EasyScript First-Strand cDNA Synthesis Kit (TransGen Biotech, China). Quantitative RT-PCR was performed with Maxima™ SYBR Green qPCR Master Mix (Fermentas, USA) on an ABI7500 real-time PCR system (Applied Biosystems, USA). Glyceraldehyde-3-phosphate dehydrogenase (GAPDH) was used for control of internal gene expression. The primers for GAPDH, L-type calcium channel α1c (Cav1.2), transient outward potassium channel (Kv4.3), MPO, MMP-2, MMP-9 and TIMP-1 are shown in Table 1.

**Table 1:** RNA primer sequences

	Forward primer	Reverse primer
GAPDH	5′-GCAAGCAGGAGTATGACGAGT-3′	5′-GGCTCTAACAGTCCGCCTA-3′
Cav1.2	5′-AGGACGCTATGGGCTATGAG-3′	5′-ACACACCGAGAACCAGATTTAG-3′
Kv4.3	5′-GACGGACTGAGACCAAACTG-3′	5′-GCTATGGAAGGAATGTTCGTG-3′
MPO	5′-CCGAGGCTACAATGACTCTG-3′	5′-GAATGTGAAGGGCTGGATG-3′
MMP-2	5′-AAGGCACATCCTACAGCAGC-3′	5′-CGAGTTCCCGCCAATAGTA-3′
MMP-9	5′-TTCGTCTTCCTGGGCAAAG-3′	5′-CTTCTTGTCGCTGTCAAAGTTG-3′
TIMP-1	5′-CTTCACCAAGACCTACGCTG-3′	5′-TCTGTCCACAAGCAATGAGTG-3′

*GAPDH* glyceraldehyde-3-phosphate dehydrogenase, *Cav1.2* L-type calcium channel α1c, *Kv4.3* transient outward potassium channel, *MPO* myeloperoxidase, *MMP* matrix metalloproteinase, *TIMP* tissue inhibitors of metalloproteinase

### Statistical analysis

Quantitative data are expressed as mean ± standard deviation. Comparisons of data before and after RAP were analyzed by *t*-test. Multiple-group comparisons were analyzed using one-way analysis of variance. SPSS 19.0 software (IBM-SPSS, Chicago, USA) was used in the statistical analysis. *P* < 0.05 is considered statistically significant.

## Results

### Electrophysiological characteristics

**Fig. 1:**
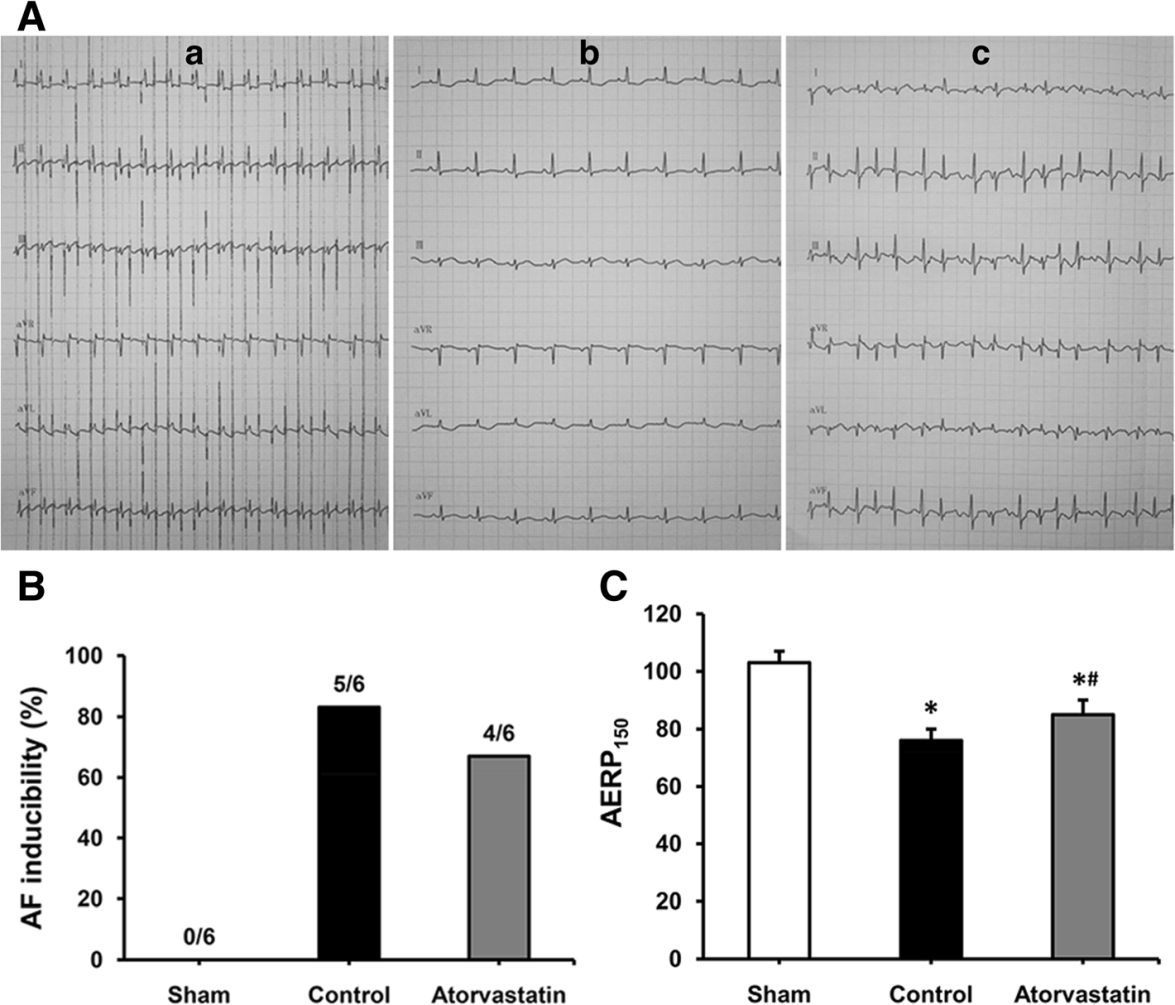
Changes in electrophysiological characteristics. **a** Representative surface ECG recordings during RAP (a), after RAP (b) and after atrial burst pacing (c) (paper speed 50 mm/s). a. ECG recordings in limb leads during RAP at 600 beats/min. b. ECG recordings in limb leads showed that the rabbit was still in normal sinus rhythm after 3 weeks of RAP when the pacemaker was deactivated. c. ECG recordings in limb leads showed that after atrial burst pacing, the rabbit was induced AF: disappearance of P-wave, and absolute irregularity of the RR interval. **b** AF inducibility in the sham, control and atorvastatin groups. **c** Comparison of AERP_150_ among the 3 groups, each bar represents the means ± standard deviation. ECG: electrocardiogram; RAP: rapid atrial pacing; AF: atrial fibrillation; AERP_150_: atrial effective refractory period at a basic cycle length of 150 ms. *P < 0.05 vs. sham group; ^#^ *P* < 0.05 vs. control group

Conventional ECGs were recorded in anesthetized intact rabbits (Fig. 1a). It is clear that 3 weeks of RAP did not cause AF, because when the pacemakers were deactivated, ECGs confirmed sinus rhythm in each rabbit in the control and atorvastatin groups.

Atrial burst stimulation induced sustained AF in 5 out of 6 (83 %) rabbits in the control group, 4 of 6 (67 %) in the atorvastatin group, but none in the sham group (Fig. 1b).

Before RAP, no significant difference in the AERP_150_ was observed among the 3 groups (sham group: 104 ms ± 5 ms, control group: 105 ms ± 5 ms, atorvastatin group: 103 ms ± 4 ms). After 3 weeks of RAP, the AERP_150_ of the control group (76 ± 4 ms) was significantly shorter than that of the sham group (103 ± 4 ms, *P* < 0.05). The AERP_150_ of the atorvastatin group was also shortened (85 ± 5 ms), but the RAP-induced reduction was reversed to some extent compare with the control group (76 ± 4 ms, *P* < 0.05) (Fig. 1c).

### Echocardiographic characteristics

Echocardiography was performed before and after RAP (Table 2). After 3 weeks of RAP, obvious LA enlargement and dysfunction was observed, but no changes in LV diameter and function were found. In the control and atorvastatin group, LAD, LAV_max_ and LAV_min_ significantly increased, whereas LAEF dramatically decreased after 3 weeks of RAP. Compared with the control group, these echocardiographic indices of LA did not differ in the atorvastatin group.

**Table 2:** Changes of echocardiographic indices before and after RAP

	LAD (mm)	LAV_max_ (ml)	LAV_min_ (ml)	LAEF (%)	LVESD (mm)	LVEDD(mm)	LVEF (%)
Sham group							
Baseline	9.73 ± 0.69	0.54 ± 0.08	0.27 ± 0.05	48.82 ± 4.48	8.46 ± 0.99	13.68 ± 1.77	72.20 ± 3.65
4 weeks post-operation	9.85 ± 0.61	0.53 ± 0.07	0.28 ± 0.05	47.59 ± 6.05	8.44 ± 0.91	13.53 ± 1.53	71.75 ± 2.81
Control group							
Baseline	9.58 ± 0.62	0.52 ± 0.09	0.26 ± 0.03	49.42 ± 3.39	8.39 ± 0.88	13.37 ± 1.23	71.18 ± 4.46
3 weeks after RAP	16.02 ± 0.84^*, **^	1.94 ± 0.28^*, **^	1.40 ± 0.18^*, **^	27.77 ± 4.18^*, **^	8.77 ± 0.64	13.35 ± 1.28	67.47 ± 2.92
Atorvastatin group							
Baseline	9.75 ± 0.56	0.55 ± 0.10	0.28 ± 0.05	49.31 ± 5.48	8.37 ± 0.94	13.58 ± 1.32	71.42 ± 3.15
3 weeks after RAP	15.53 ± 0.62^*, **^	1.78 ± 0.24^*, **^	1.29 ± 0.17^*, **^	27.32 ± 3.70^*, **^	8.78 ± 0.78	13.64 ± 1.22	68.50 ± 5.34

Data are reported as mean ± standard deviation

*RAP* rapid atrial pacing, *LAD* left atrial diameter, *LAV*_*max*_ left atrial maximal volume, *LAV*_*min*_ left atrial minimal volume, *LAEF* left atrial ejection fraction, *LVESD* left ventricular end systolic diameter, *LVEDD* left ventricular end diastolic diameter, *LVEF* left ventricular ejection fraction

^*^ *P* < 0.05 vs. baseline

^**^ *P* < 0.05 vs. sham group

### Atrial structural remodeling

**Fig. 2:**
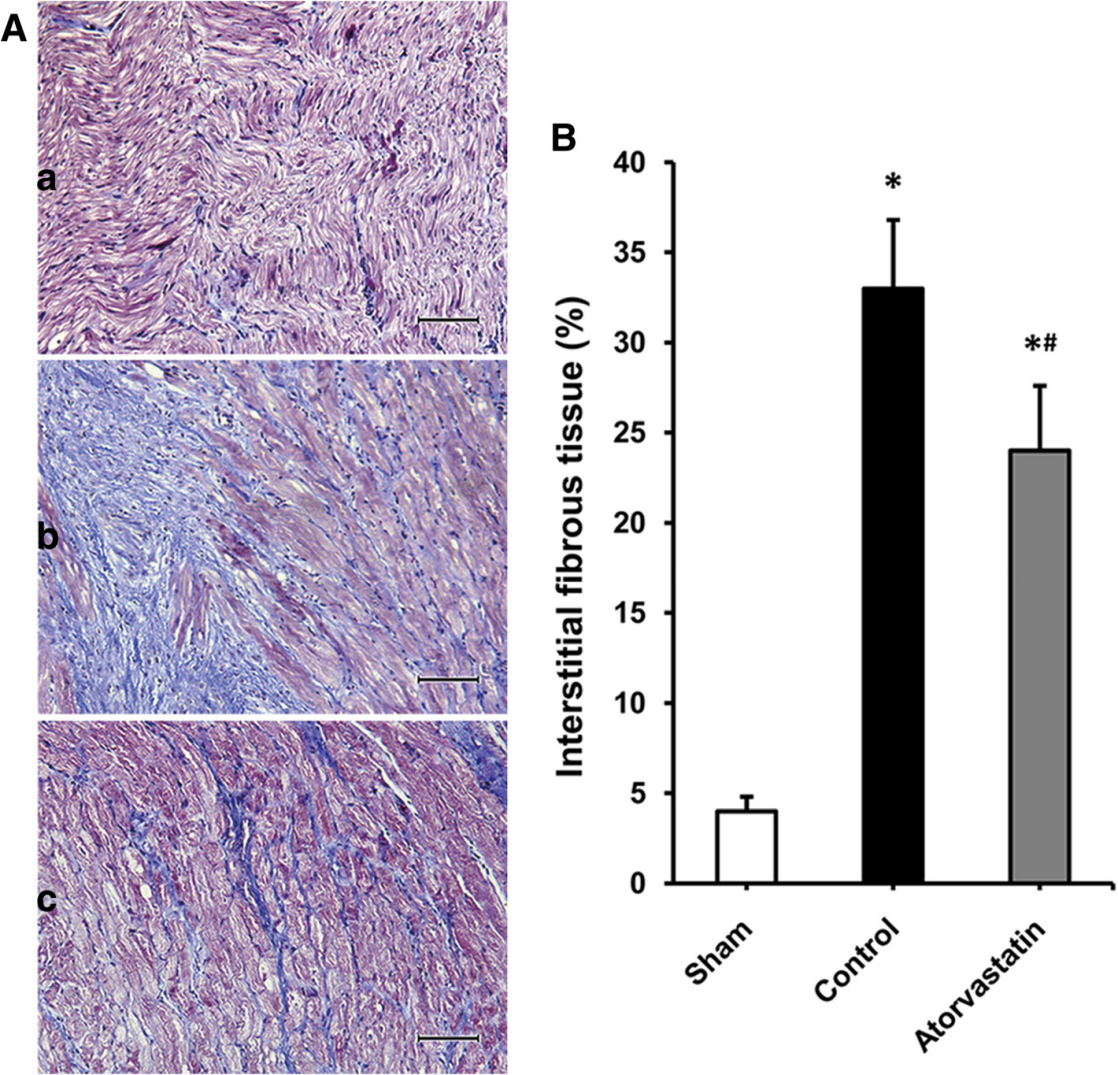
Histological analysis of atrial interstitial fibrosis. **a** Representative Masson trichrome-staining of left atrial myocardium in the sham (a), control (b) and atorvastatin (c) groups (The magnification is × 200, scale bar: 50um). **b** Percentage of areas of interstitial fibrous tissue among the 3 groups, each bar represents the means ± standard deviation. *P < 0.05 vs. sham group; ^#^ *P* < 0.05 vs. control group

As shown in Fig. 2, RAP caused a marked LA interstitial fibrosis as estimated by Masson trichrome-staining. The degree of atrial fibrosis in the control and atorvastatin group was significantly higher than that in the sham group. Compared with the control group, the atrial fibrosis was partial suppressed in the atorvastatin group.

**Fig. 3:**
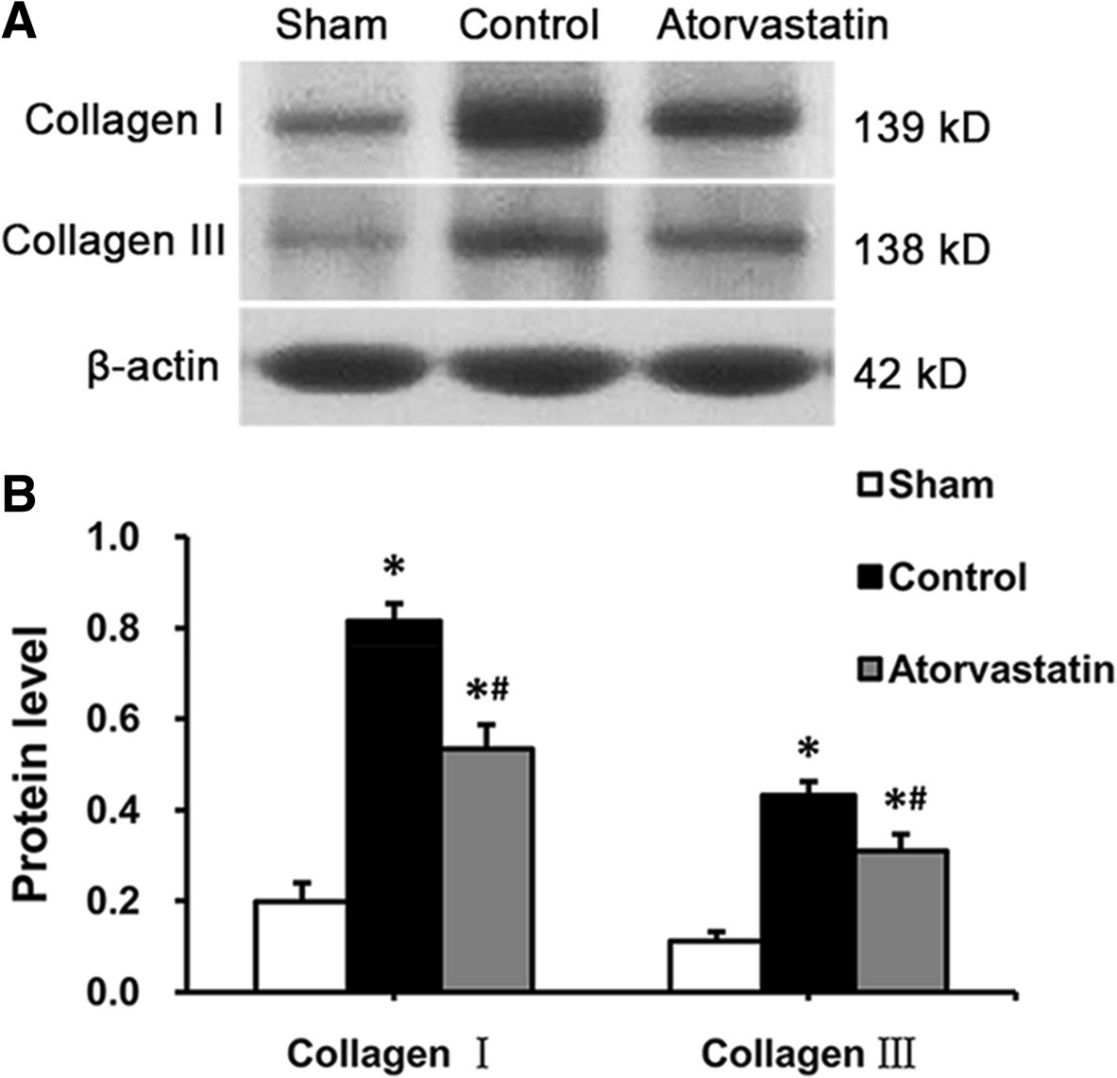
The protein levels of collagen I and collagen III in the left atrium. **a** Representative western blot gels depict the protein expression levels of collagen I and collagen III. **b** Mean values of the protein expression levels of collagen I and collagen III in the 3 groups. Each bar represents the means ± standard deviation. *P < 0.05 vs. sham group; ^#^ *P* < 0.05 vs. control group

As shown in Fig. 3, the protein levels of collagen I and collagen III were significantly increased in the control group in comparison with the sham group. These changes in the levels of these 2 types of collagen were suppressed by atorvastatin.

### Atrial ion-channel remodeling

**Fig. 4:**
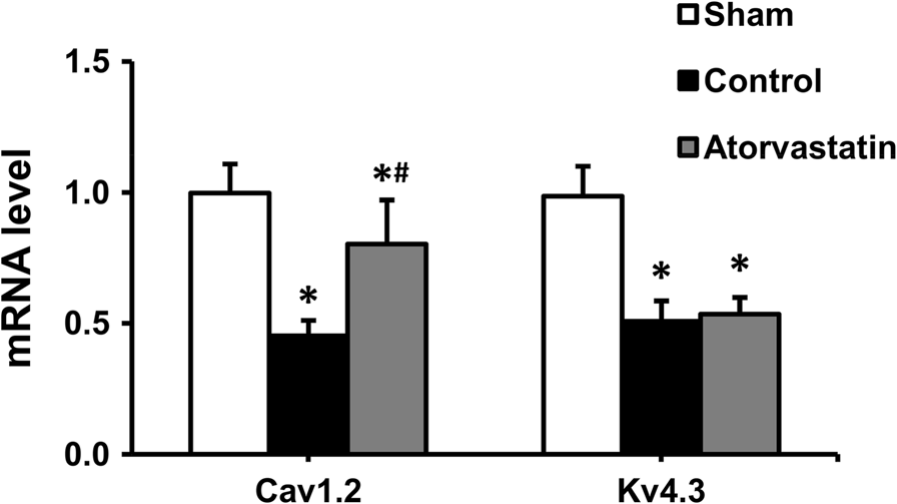
The mRNA levels of Cav1.2 and Kv4.3 in the left atrium. Each bar represents the means ± standard deviation. Cav1.2: L-type calcium channel α1c; Kv4.3: transient outward potassium channel; mRNA: micro ribonucleic acid. *P < 0.05 vs. sham group; ^#^ *P* < 0.05 vs. control group

As shown in Fig. 4, the Cav1.2 mRNA significantly decreased in the control group compared with the sham group, but it down-regulation was prevented in the atorvastatin group. The Kv4.3 mRNA was also significantly decreased in the control group compared to the sham group, but it down-regulation was not prevented in the atorvastatin group.

### Levels of MPO, MMP-2, MMP-9 and TIMP-1 in the LA

**Fig. 5:**
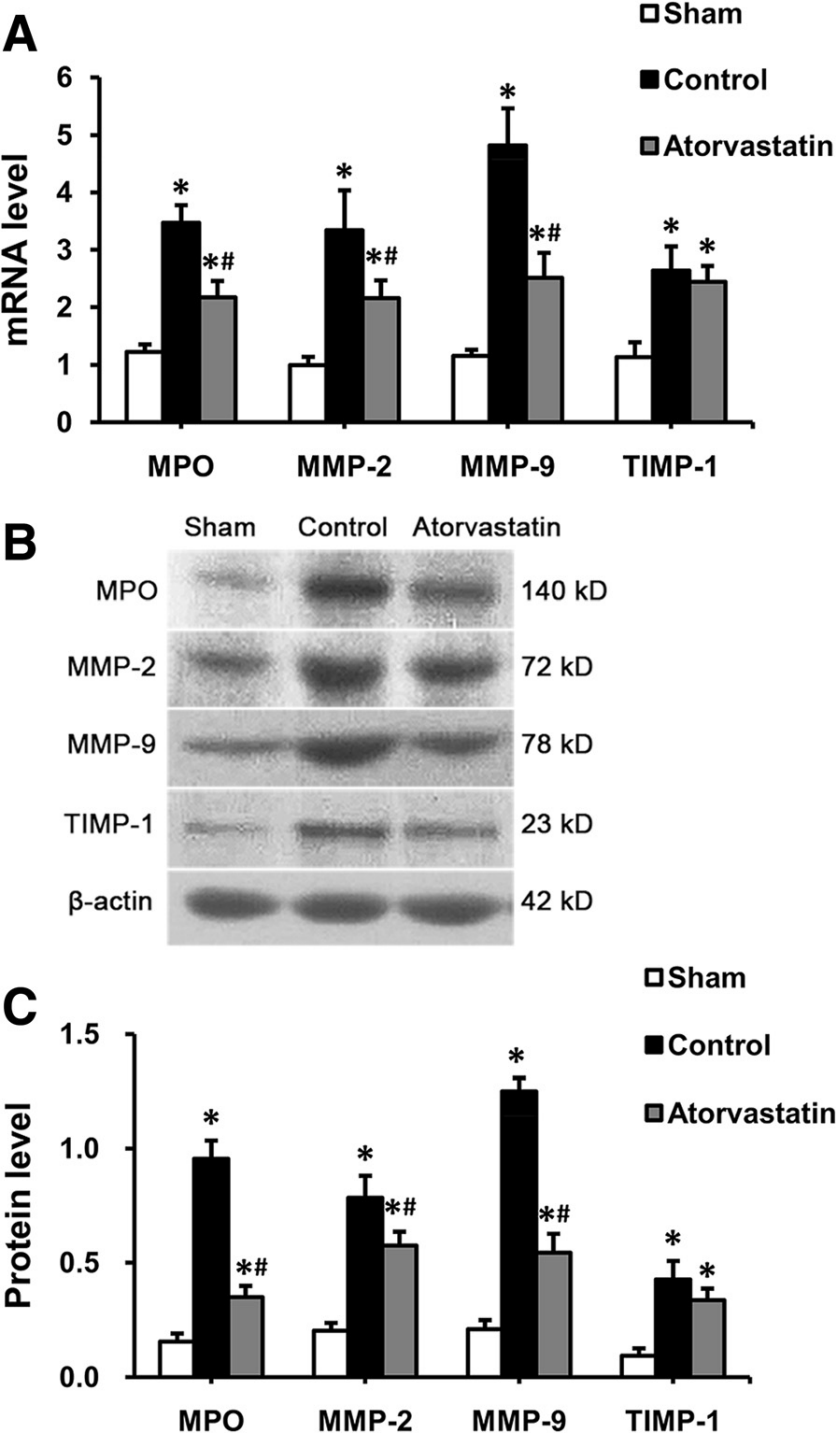
The levels of MPO, MMP-2, MMP-9 and TIMP-1 in the left atrium. **a** Mean values of the mRNA expression levels of MPO, MMP-2, MMP-9 and TIMP-1 in the 3 groups. **b** Representative western blot gels depict the protein expression levels of MPO, MMP-2, MMP-9 and TIMP-1. **c** Mean values of the protein expression levels of MPO, MMP-2, MMP-9 and TIMP-1 in the 3 groups. Each bar represents the means ± standard deviation. mRNA: micro ribonucleic acid; MPO: myeloperoxidase; MMP: matrix metalloproteinase; TIMP: tissue inhibitors of metalloproteinase. *P < 0.05 vs. sham group; ^#^ *P* < 0.05 vs. control group

As shown in Fig. 5, the mRNA and protein levels of MPO, MMP-2 and MMP-9 were significantly increased in the control group compared with the sham group. These changes in the levels of MPO, MMP-2 and MMP-9 were suppressed by atorvastatin. The level of TIMP-1 was also increased in the control group compared to the sham group, but atorvastatin treatment did not suppress the up-regulation of TIMP-1.

## Discussion

The present data demonstrated that in the rabbit model of RAP-induced AF, atorvastatin suppressed AERP shortening and atrial interstitial fibrosis induced by RAP, but had no effect on RAP-induced atrial enlargement and dysfunction. In addition, atorvastatin suppressed the down-regulation of Cav1.2 mRNA, and prevented the increase in the levels of collagen I and III, MPO, MMP-2 and MMP-9 induced by RAP.

The main mechanisms contributing to AF initiation and maintenance are atrial electrical and structural remodeling. In recent years, several animal models of AF [11–13, 20, 21] have been developed to investigate the molecular mechanism contributing to atrial remodeling. Among them, the dog [11, 14, 20] and rabbit [17, 19, 21] AF models induced by RAP are widely used. However, if atrioventricular block is not performed, dogs will develop significant LV dysfunction induced by RAP [20], whereas rabbits will not [21]. It is well known that LV dysfunction will subject the LA to a pressure overload, leading to atrial enlargement and electrical instability [22]. Therefore, in the present study we chose rabbits to create an AF animal model to avoid the influence of LV dysfunction on atrial remodeling.

### Effects of atorvastatin on atrial structural remodeling

Atrial structural remodeling is characterized by atrial enlargement and interstitial fibrosis [4, 23], and has been considered as a major contributor to AF [23]. LA enlargement has been identified as an independent risk factor for AF. For example, patients are more prone to paroxysmal AF if they have an increased LAD [24]. Larger LA volume before cardioversion is associated with higher risks of AF recurrence [25]. LA enlargement also significantly correlates with atrial fibrosis, which serves as a crucial substrate in the formation of AF and is difficult to reverse [26]. Increased fibrosis has been observed in the atrium of animal models [20, 21] and patients with AF [27]. It is characterized by enhanced deposition of matrix collagen proteins, leads to inhomogeneous atrial electrical conduction, and gives rise to electrical reentry circuits which result in AF [6].

In our study, after 3 weeks of RAP, rabbits showed significant atrial structural remodeling. The pacing time of our rabbit AF model is relatively short compared with the previous canine AF model [14, 20], but rabbits have already had obvious atrial enlargement and interstitial fibrosis. In the previous canine AF model, after 4–6 weeks of RAP, LA volumes were nearly 2 times that at baseline [14, 28], and atrial fibrosis of the control group was nearly 9–10 times that of the sham group [20, 28]. In our model, after 3 weeks of RAP, LA volumes were nearly 4 times that at baseline, and atrial fibrosis of the control group was nearly 7 times that of the sham group. The obvious atrial structural remodeling contributed to a marked increase in AF inducibility. After 3 weeks of RAP, although all rabbits in the control group remained sinus rhythm when pacemakers were deactivated, atrial burst pacing induced sustained AF almost in all rabbits.

Our study showed that atorvastatin treatment could not prevent AF susceptibility and atrial enlargement and dysfunction, but could prevent atrial interstitial fibrosis and collagen protein expression levels. These results are not completely consistent with the previous research [14], which showed that statins could not prevent AF susceptibility, but could prevent atrial dilatation and fibrosis in a canine AF model induced by 6 weeks of RAP. The different effects of statins on atrial dilatation may be attributed to different AF animal models and drug intervention time. In addition, LA volumes after RAP were only 2 times that at baseline in the previous research, while in our study these were nearly 4 times that at baseline, which may predict more serious LA enlargement, so is hard to reverse.

The metabolism of extracellular matrix is regulated by MMPs and their inhibitors, the TIMPs [29]. Among many kinds of MMPs and TIMPs, MMP-2 and MMP-9 are key factors leading to atrial fibrosis in AF [5, 30, 31], while TIMP-1 is a major inhibitor of MMP activity in AF tissues [31]. MPO, a major contributor to inflammatory oxidative stress, also has an important role in AF. It could promote MMP expression and activation by catalyzing the generation of reactive species, and subsequently resulted in atrial fibrosis and AF [5, 6]. Previous research showed that patients with AF had higher plasma and atrial MPO levels compared with individuals in sinus rhythm [5], and high MPO levels predicted an increased risk of AF recurrence after catheter ablation [7]. In addition, MPO-deficient mice were protected from atrial fibrosis and AF vulnerability induced by angiotensin II, and atrial MMP-2 and MMP-9 levels were profoundly reduced. However, if administrated with recombinant MPO, these MPO-deficient mice would develop a similar degree of atrial fibrosis as that observed in MPO-infused wild type mice [5].

Many studies showed that statins, by their anti-inflammatory and antioxidant properties, could reduce the levels of plasma MPO in patients with cardiovascular diseases [15, 16], and inhibit MPO mRNA expression in macrophages [32]. In addition, statins also could inhibit secretion of MMP-2 and MMP-9 [33], and down-regulate their expression levels [34, 35]. In our study, the levels of MPO, MMP-2, MMP-9 and TIMP-1 were significantly increased after RAP. Atorvastatin treatment could suppress the increased levels of MPO, MMP-2 and MMP-9, especially MPO and MMP-9, but could not suppress the increased level of TIMP-1. These may be the potential mechanisms by which statins prevent atrial structural remodeling of AF.

In addition, peroxisome proliferator-activated receptor-gamma (PPARγ) is also involved in atrial remodeling and AF. Recent studies showed that PPARγ was decreased in elderly AF patients [36] and hypertensive AF patients [37], while PPARγ agonists could inhibit atrial remodeling in AF models [38, 39] and prevent new onset AF in patients with non-insulin dependent diabetes [40]. Statins could activate PPARγ and enhance its expression [41, 42] by their anti-inflammatory and antioxidant properties. Therefore, whether the modulation of statins on PPARγ is involved in the molecular mechanisms of the prevention of statins against atrial remodeling in our rabbit model of AF is still a question and would be investigated in our future study.

### Effects of atorvastatin on atrial electrical remodeling

Atrial electrical remodeling is characterized by ion channel dysfunction [4], which creates a re-entry-prone substrate. In our study, 3 weeks of RAP caused AERP shortening and down-regulation of Cav1.2 and Kv4.3 mRNA. This is consistent with previous research using dog AF models [11, 20, 21]. In the present study, atorvastatin treatment could partially suppress AERP shortening and Cav1.2 mRNA down-regulation, but had no effect on Kv4.3 mRNA down-regulation. Many studies have proved that atrial electrical remodeling was promoted by inflammation and oxidative stress, while could be reversed by statins [8, 11, 13, 43]. As mentioned above, atorvastatin treatment suppressed the increased level of MPO, which is a major contributor to inflammatory oxidative stress. Therefore, our study suggests that statins may prevent electrical remodeling, and the reduced atrial MPO level may contribute to the prevention of statins on this process.

## Conclusion

The present study demonstrated that atorvastatin treatment prevented atrial remodeling in a rabbit model of RAP-induced AF. The reduction of levels of atrial MPO, MMP-2 and MMP-9 may contribute to the prevention of atorvastatin on atrial remodeling. These findings provide pharmacological evidence for the clinical use of statins in the treatment of AF.

## Limitations

The sample size was relatively small, and the duration of RAP was relatively short. In this study, we only measured the levels of MPO, MMP-2 and MMP-9, but did not measure their enzymatic activity in the atrium. In addition, we did not investigate whether the preventive effects of atorvastatin on atrial remodeling of AF were dose-dependent, and did not conduct detailed molecular study in cardiac tissue as well as extracellular matrix remodeling due to some methodological limitations. Last but importantly, we did not show the causality between the inhibition of MPO by statins and the suppressed atrial remodeling. MPO might be just a concomitantly induced factor rather than a key mediator in our model.

## Abbreviations

AERP, atrial effective refractory period; AF, atrial fibrillation; Cav1.2, L-type calcium channel α1c; ECG, electrocardiogram; GAPDH, glyceraldehyde-3-phosphate dehydrogenase; Kv4.3, transient outward potassium channel; LA, left atrium; LAEF, left atrial ejection fraction; LAV_max_, left atrial maximal volume; LAV_min_, left atrial minimal volume; LV, left ventricle; LVEDD, left ventricular end diastolic diameter; LVEF, left ventricular ejection fraction; LVESD, left ventricular end systolic diameter; MMP, matrix metalloproteinase; MPO, myeloperoxidase; PPARγ, peroxisome proliferator-activated receptor-gamma; RAP, rapid atrial pacing; RT-PCR, real time polymerase chain reaction; TIMP, tissue inhibitors of metalloproteinase
